# Pharmacy response to COVID-19: lessons learnt from Canada

**DOI:** 10.1186/s40545-020-00280-w

**Published:** 2020-12-09

**Authors:** Ali Elbeddini, Amy Botross, Rachel Gerochi, Mohamed Gazarin, Ahmed Elshahawi

**Affiliations:** 1 Winchester District Memorial Hospital, 566 Louise Street, Winchester, ON KK0C2K0 Canada; 2 Leslie Dan Faculty of Pharmacy, University of Toronto, 144 college st, Toronto, M5S 3M2 Canada; 3 1 King’s College Circle, Toronto, ON M5S1A8 Canada

**Keywords:** COVID-19, Opportunities, Wave-2

## Abstract

When the first wave of COVID-19 hit in March 2020, health care professionals across Canada were challenged to quickly and efficiently adapt to change their work practices in these unprecedented times. Pharmacy professionals, being some of the very few front-line health care workers who remained accessible in person for patients, had to rapidly adopt critical changes in their pharmacies to respond in the best interest of their patients and their pharmacy staff. As challenging and demanding as such changes were, they provided pharmacists with invaluable lessons that would be imperative as the country enters a potentially more dangerous second wave. This article seeks to identify and summarize opportunities for improvement in pharmacy as learnt from the pandemic’s first wave. Such areas include but are not limited to handling of drug shortage and addressing drug hoarding and stockpiling, providing physical and mental support for staff, timing of flu vaccine and COVID-19 screening/testing, collaboration between different health care sites as well as collaboration with patients and with other health care professionals, telemedicine and willingness to adopt innovative ideas, need for more staff training and more precise research to provide accurate information and finally the need for more organizational and workplace support. Learning from what went well and what did not work in the early stages of the pandemic is integral to ensure pharmacy professionals are better prepared to protect themselves and their patients amidst a second and possibly subsequent waves.

## Background

When COVID-19 pandemic started, pharmacy professionals found themselves facing multiple challenges that surfaced during these unprecedented times and were forced to act quickly and safely to protect their patients and their staff. As the pandemic’s first wave progressed and started to peak, the country’s medical clinics and physicians’ offices closed their doors to the public and opted for virtual patient-interactions as a strategy to mitigate infection spread. Pharmacists quickly became some of the very few health care professionals (HCPs) accessible in person to the general public to provide medical advice. They efficiently worked tirelessly on the frontlines to provide additional services in an attempt to ease the burden of the healthcare system and prevent unnecessary visits to healthcare facilities [1]. Many pharmacies also initiated “Drive thru” services to optimize safety of both patients and staff [2]. Given the unparalleled work that pharmacists are now doing on the front lines, it would be hard to believe that even before COVID-19 hit, there has been a long ongoing debate of whether Canadian pharmacists are considered front-line HCPs. They have even been left out from Ontario’s own list of front-line workers who were eligible for the 16-week pandemic pay from April 24, 2020 until August 13, 2020 [3].

Pharmacists’ key responsibilities during COVID-19 shifted dramatically, in such a short period of time, to include delivery of pharmacists’ services through telehealth and telemedicine. As COVID-19 impacted direct patient care, telemedicine provided an opportunity to optimize medication management for the high-risk immunocompromised population and for people who were self-isolating [4]. Pharmacists also became increasingly involved in managing and renewing chronic medications, providing minor ailment consultations, clarifying misconceptions and screening patients for COVID-19 symptoms [5]. Moreover, pharmacists spend a significant amount of time communicating and connecting with other HCPs, on behalf of their patients, to clarify drug orders and offer recommendations and pharmaceutical opinions to enhance medication management. Another challenge that pharmacists had to deal with was the repeated drug shortages of essential medications such as salbutamol metered-dose inhalers (MDI), azithromycin and hydroxychloroquine [6]. Pharmacists were, thus, regularly monitoring the drug supply chain to anticipate drug shortages and ensure patients were advised against hoarding or stockpiling of medications [7]. All the aforementioned responsibilities have undoubtedly added stress and anxiety to the pharmacist’s workday [8]. Pharmacists had to quickly learn to manage this highly unsafe and stressful situation, with little support provided and insufficient personal protective equipment [9]. They struggled to ensure their own mental well-being and safety as well as the safety of their immediate family members and friends.

**Fig. 1:**
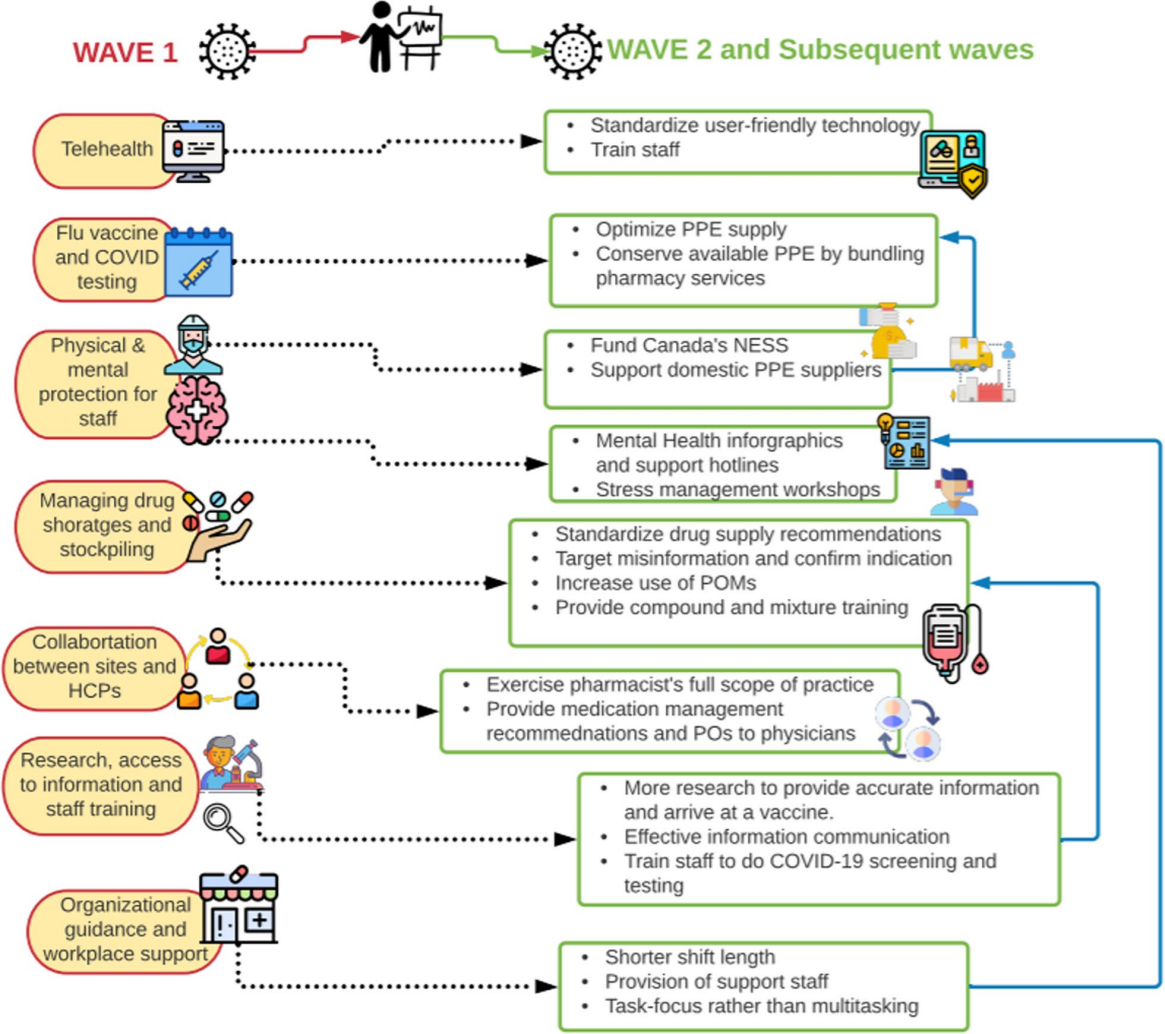
Potential areas of improvement in pharmacy as learnt from COVID-19 wave 1

There is, thus, no doubt that pharmacy professionals have prioritized their patients and stepped-up to the front lines during the pandemic’s first wave to provide valuable and much needed care to the public. However, multiple challenges and difficulties have risen in the pharmacy field and it is crucial that pharmacy professionals study and learn from these shortcomings so that they are better equipped for the pandemic’s subsequent waves (Fig. 1).

## Discussion

### Telemedicine and adoption of technology and innovative practices

There is no doubt that telehealth and technology played an essential role in patient care provision during the early stages of the pandemic. Telehealth ensured that high-risk patients, such as the elderly and the immunocompromised, could still access healthcare while at home. Such technologies included virtual phone or zoom consultations and medication reviews, central fill systems and well as phone/online appointment booking and refill requests. Though this enhanced uptake of technology and virtual platforms helped make workflow more efficient and increased the safety of pharmacy staff and patients, it also came with another challenge as many pharmacy staff struggled to navigate through it. Pharmacies that had adopted some forms of technology prior to the pandemic were much better prepared to operate when COVID-19 hit; however, other pharmacies struggled to keep up with the learning curve of the new technologies [9].

This serves as a good future opportunity for investment in incorporating standardized communication technologies and dispensing software in pharmacies. Standard technology will make it easier for staff to use regardless of where they practice. Technologies that are chosen should be user-friendly, time-efficient, and easy for the staff of all ages to understand. Training sessions should also be provided to all staff to educate them on how to integrate these technologies in daily tasks, how to troubleshoot, and how to use such platforms to save time and effort and enhance workflow efficiency.

### Providing physical and mental protection/support for pharmacy staff

During the early stages of the pandemic, basic personal protective equipment (PPE) such as masks, N95 respirators, gloves, gowns and face shields, became crucial to ensure safety of front-line staff. There was, however, a significant delay in supplying HCPs with the necessary equipment and sufficient PPE inventory as the country scrambled to meet this unprecedented demand [10, 11]. As a result, pharmacists often found themselves providing direct patient care without adequate PPE, which threatened their own personal safety. Such critical shortage has prompted Ontario Health to release, and regularly update, recommendations and guidelines about optimizing the supply of PPE during the pandemic [12]. It is thus not surprising that the need for enhancing PPE supply and increasing pharmacy-based security became common themes during the pandemic’s first wave [9]. One main reason behind this PPE crisis is the fact that Canada’s National emergency stockpile system (NESS) was completely unprepared for such an event due to decades of underfunding [13]. Another main reason was the country’s dependence on two main foreign PPE suppliers, the US and China, whose supply chains became hugely unreliable during the pandemic [13]. Moving forward, Canada’s NESS should be sufficiently funded in order to be prepared to supply the country’s front-line workers with the necessary equipment in anticipation of future COVID-19 waves or similar outbreaks. Moreover, funds should be allocated to establish and support domestic PPE suppliers that could be relied on whenever foreign trade is interrupted.

Aside from heightened physical stresses, the early stages of the pandemic also brought mental distress to pharmacists, and other medical staff, as depression, anxiety and insomnia became the most prevalent mental health disorders among health care workers during the pandemic’s first wave [14]. Pharmacists, in particular, have noticed an increase in their workload as their roles amid COVID-19 shifted to include virtual consultations, patient triaging, COVID-19 screening, information dissemination and medication shortage management, all of which have significantly impacted their mental health. Such mental stressors were also echoed by pharmacy professionals in many commonwealth countries as they reported general extreme worry and anxiety about the personal and professional impact of the pandemic [15]. Harassment and under-appreciation by the public and government during the pandemic also added further burden to pharmacists' psychological well-being. Moving forward, mental health support resources should be made available 24/7 to front-line workers, including pharmacists, to ensure mental stresses are being addressed. Such resources could include infographics, such as the one generated by CPhA that provide information for pharmacists on how to effectively manage workload as well as guide them to provincial resources [16]. Other forms of mental health support can also include hotlines that are accessible to HCPs 24/7 so they can chat and share their experiences, anonymous mental-health helplines and stress-management workshops where HCPs can get together to share and learn new stress-management strategies.

### Timing of flu vaccines with COVID screening/testing in community pharmacies

As the bulk of the second wave of COVID-19 approaches, there is the additional worry of influenza season. Although there is evidence demonstrating that measures used to control the spread of COVID-19 decreases influenza activity substantially, there are still reports of co-infection [17–19]. This co-infection has the potential to complicate prognosis and is especially concerning in high-risk groups such as older individuals, those with chronic diseases and the immunocompromised [20, 21]. A high uptake of influenza immunizations is therefore an important component in preparing for the next wave of COVID-19. With the introduction of high-dose influenza vaccines in community pharmacies in Ontario, the number of influenza vaccines to be administered is anticipated to be much larger compared to previous years. The pandemic has also increased public health awareness and has stressed the importance of vaccines to proactively prevent illness, also potentially contributing to the rising demand of vaccines. Therefore, influenza season, together with the launch of asymptomatic testing, will result in a very high volume of traffic in Ontario community pharmacies. In light of personal protective equipment (PPE) and disinfectant shortages, it is important to develop strategies to conserve pharmacy supply of these products as well as minimize COVID-19 exposures for both staff and patients visiting the pharmacy.

A strategy to address these concerns would be to bundle as many pharmacy services as possible together during patient visits. By building services such as vaccine administrations, asymptomatic testing, and medication pick-up where insurance permits, reduces multiple pharmacy visits and exposure. This ultimately saves PPE and disinfectant related costs and reduces the amount of times pharmacy staff must complete a thorough cleaning between patients. Providing these services furthermore maximizes patient care during the pandemic by not only increasing influenza vaccination uptake but also ensuring completion of any other outstanding vaccinations. This is especially helpful for patients when many doctors are only providing phone consultations in the meantime. There have been concerns revolving around the influenza vaccine’s potential to cause false positives for patients tested for COVID-19 and to reduce patients’ ability to fight off the infection [22]. As of now, there is no evidence proving these claims to be true but there is some evidence supporting the opposite. It was found that in areas of Italy where there were higher rates of influenza vaccination, there was an overall lower rate of mortality from COVID-19 [23]. Furthermore, by reducing the number of influenza cases during the pandemic, less individuals will require COVID-19 confirmatory testing when presenting with influenza symptoms. Although this will not get rid of the current testing backlog, it can help reduce unnecessary testing [24].

### Handling of drug shortages, hoarding and stockpiling

Not only was there a concern with the shortage of PPE, medication shortages were also rampant during the first wave of the coronavirus pandemic, affecting pharmacists across different levels of care [25]. The cause of these shortages is multifaceted and therefore has to be addressed as such in order to preserve the drug supply and avoid barriers to care due to insufficient resources [26]. To tackle this issue from the community pharmacy perspective, the Ministry of Health recommended pharmacies to limit patient medication supplies to a maximum of 30 days except in special circumstances based on pharmacist judgement. This recommendation was established to prevent stockpiling and to guarantee all patients have access to their medications [27]. Despite this call for pharmacies, it still remained a “recommendation” and it is unknown whether all pharmacies were compliant, especially due to pushback from patients and the delayed enactment of ODB dispensing fee compensation [28]. Some pharmacies responded to the pushback by reducing their dispensing fee so that patients would not incur additional costs due to the 30-day limitation, however, some pharmacies do not have the funds to absorb this cost especially with the additional provision of free deliveries. In preparation for the second wave of COVID-19, there must be a balance between the costs to pharmacies and patients so as to not jeopardize medication compliance due to increased costs, particularly during a time where many individuals have lost their jobs. Acceptance of this recommendation may be increased in the coming months as patients have already navigated these requirements and easy implementation of dispensing fee coverage by ODB. It may be of value for private insurance companies to investigate strategies to compensate for the increase in dispensing fee costs for essential medications. Not only was there potential for inconsistencies of the 30-day limit between pharmacies, there were many inconsistencies in this recommendation across provinces. Although most provinces called for the 30-day medication limit, many lifted the limitation at different times, with some provinces lifting the limit as early as April and May [29]. The drug supply is shared across Canada and therefore, the efforts to conserve the supply should take place uniformly across Canada as well. Through the combined efforts of provincial health bodies and stricter limitation policies, the impact towards the conservation of medications is more pronounced.

The spread of misinformation such as the efficacy of certain medications in the treatment of COVID-19 such as hydroxychloroquine has also contributed to medication shortages [30]. As medication experts, it is important for pharmacists to evaluate prescriptions for these medications to ensure their appropriate use and to maintain patient safety. This expertise may also be applied to other medications that were affected by the pandemic such as salbutamol pressurized metered-dose inhalers (pMDIs). Many COVID-19 patients received salbutamol pMDI as other dosage forms such as nebulized salbutamol increased risk of droplet contamination. Pharmacists may work together with the patient to ensure they are compliant with their maintenance therapy that often accompanies salbutamol use to minimize overall need for their salbutamol pMDI. Pharmacists may also recommend alternate dosage forms that are available, for example the Diskus format. Where there are no alternatives and treatment is considered essential, a combination product may be warranted [31].

A strategy to prevent or conserve the drug supply chain within hospitals is increasing the use of patient’s own medications (POMs). When admitted, oftentimes patients’ medications are automatically substituted to the hospital formulary equivalent. If POMs are obtained whenever possible, medication wastage is reduced, even more so if the hospital does not have to obtain a non-formulary medication for a specific patient. This is also helpful in cases where manufacturer package size is beyond what the patient requires for their treatment for example for pMDIs and topicals [32]. An initiative known as the “Green Bag Scheme” has been implemented in United Lincolnshire Hospital pharmacy teams in order to facilitate POM use within hospitals if appropriate. Green bags are supplied to patients to collect medications that a patient requires if they need to go to the hospital. All medications, as well as new medications, are kept together inside this bag and follow the patient’s hospital transitions [33]. Implementation of a similar system may be helpful in reducing overall medication waste that may potentially contribute to drug shortages. Of note, the medication supply for the critically ill patients has been significantly impacted by COVID-19 especially sedatives and neuromuscular blocking agents. Some strategies that have been used include using adjunctive oral medications to limit the use of medications in short supply or supplementing IV formulations with its oral or transdermal form [34]. A hospital in New York City addressed shortages of these particular medications through compounding larger product sizes or higher concentrations to enable longer administration times. A potential downside to this method is the shortened expiration period of a week, however, this particular hospital also implemented beyond-use dating per US guidelines and regulations. As a preparation for the next wave of COVID-19, a call for more prepackaged or premixed IV medications is vital in order to lengthen the administration expiry dates of these products. This furthermore encouraged intermittent bolus dosing of neuromuscular blocking agents where possible. If this schedule was not feasible, the lowest dose was targeted in order to achieve ventilator synchrony [35]. Alternatively, pharmacy staff can be trained to compound specific medications or mix IV medications that are in high demand but undersupplied in order to ensure they are promptly ready on site when needed.

### Collaboration between sites, healthcare professionals (HCPs) and patients

With the overall decrease of access to in-person physician visits, the role of a pharmacist grows as they remain one of the most accessible health care professionals in the community during this time. With this in mind, collaboration between pharmacists, physicians and patients becomes very important to maximize patient care. By providing over the counter (OTC) recommendations to patients and medication management recommendations to physicians, pharmacists can help provide care to patients while relieving virtual physician appointments so that patients in more urgent need can receive help in a more timely manner. Exercising a pharmacist’s full scope of practice such as extending chronic medications where appropriate or adapting a prescription can also alleviate COVID-19 related healthcare backlog. There is also no question that the pandemic has been related to rising cases of mental illness around the world [36]. Due to their accessibility, pharmacists can furthermore collaborate with patients to direct them to mental health resources that they can access.

### Research, access to accurate information and staff training

The pandemic panic that ensued earlier gave rise to many questions and concerns from the public. As the public’s first point of contact for information and the most accessible health care professionals, pharmacists were expected to provide accurate information in a timely manner. There was, and still is, a dire need for good-quality research as identified by International Pharmaceutical Federation (FIP) Pharmacy Practice Research Special Interest Group to educate pharmacists and inform their practice [37]. During the pandemic pharmacists’ took on new responsibilities without prior training or preparation, which included conducting COVID-19 screening, handling suspected COVID-19 cases and finally most recently conducting COVID-19 testing [38].Though pharmacy staff were able to show their resilience and ability to adapt as front-liners, there were definitely some struggles and challenges along the way. Such struggles can be attributed mainly to lack of research and information as well as lack of training on these new services. Pharmacists, like the rest of the population, came across a lot of conflicting evidence about important topics such as the proposed roles of ibuprofen and hydroxychloroquine in COVID-19. Pharmacists reported the need for more guidance and access to reliable information repositories to help address evolving pandemic-related questions. There is, thus, an apparent need for more research to arrive at accurate information that pharmacists can access and relay to the public [9].

Another issue that arose was the way information was being communicated. Although emails were regularly being sent out by regulatory bodies and national associations, and websites were regularly updated, there was a reported lack of awareness of such tools and claims of ambiguous communication methods [39]. There is thus a need to raise awareness of the available information sources as well as employ appropriate communication tools that are easy for pharmacists to access.

Research should not only be focused on merely studying the characteristics of the novel virus, but efforts should also be geared towards finding an effective vaccine. Although there are currently many vaccine candidates in different clinical trial stages, there has not yet been any successful candidates that are safe and effective to be administered [40]. Research efforts should thus be intensified in this area.

### Need for organizational guidance and workplace support

Little organizational guidance and workplace support was also another issue that pharmacists faced during the early stages of the pandemic. Canadian community pharmacists reported that although they relied heavily on information provided by regulatory bodies and professional associations to provide patient care during the pandemic, they are dis-satisfied by how such information was communicated and would have liked more direction from their corporates and workplaces [39].

A study by Austin and Gregory that investigated the experience of Canadian community pharmacists during the early stages of the pandemic identified “organizational resilience” as a common theme that emerged during the first wave [39]. Pharmacists called for the need for more practical guidance provided by their employers on how to deal with evolving workplace pandemic-related issues. The study identified three workplace strategies that pharmacists reported would be more helpful in increasing resilience during the pandemic. The first strategy is a workplace and a management team that highlights task-focus and reduces multitasking as this provides pharmacy staff with a greater capacity to effectively manage workload. The second strategy is shortened shift lengths and mandatory short breaks for pharmacy staff as these mental and physical breaks are essential in ensuring the safety of workflow. Lastly, pharmacists called for the need of more support staff in their workplaces to carry out non-professional tasks such as ensuring physical distancing in the store, ensuring visitors have their masks on before approaching the pharmacy counter, conducting deep cleaning and disinfection protocols as well as security staff to handle violent patients. Pharmacists have reported that they have felt less physical and mental stress when their workplaces provided extra workers which helped them focus on clinical tasks [39].

That being said, given Canada’s solid pharmacy organizational culture as well as the deep understanding of its “personality” and cultural attributes [41], pharmacy has been able to effectively adapt and change, as an organization, during the pandemic’s first wave. This same strong cultural framework can also be applied to facilitate future efforts and changes within pharmacy to prepare for subsequent outbreaks.

## Conclusion

It is no doubt that the first wave of COVID-19 in Canada proved to be an unprecedented challenge to pharmacy professionals and patients. As the brunt of the second wave of COVID-19 approaches, it is important to identify both areas of strengths and weaknesses from the management of the first wave of the virus in an effort to decrease excessive strain on the healthcare system that can be detrimental to the delivery of patient care. There are key areas to focus on that are of particular interest from the first wave. These include preventing and confronting drug shortages, ensuring effective collaboration between health care teams and patients, workplace support, improved training and education as well as implementation of technology to facilitate patient care. Focusing on these key areas to employ strategies learned from the first wave together with lessons learned from institutions around the world will be vital to ensuring that pharmacists are able to maintain their ability to provide effective care.

## Data Availability

Data sharing does not apply to this article as no datasets were generated or analyzed during the current study.
